# Disparities in availability of essential medicines to treat non-communicable diseases in Uganda: A Poisson analysis using the Service Availability and Readiness Assessment

**DOI:** 10.1371/journal.pone.0192332

**Published:** 2018-02-08

**Authors:** Mari Armstrong-Hough, Sandeep P. Kishore, Sarah Byakika, Gerald Mutungi, Marcella Nunez-Smith, Jeremy I. Schwartz

**Affiliations:** 1 Epidemiology of Microbial Diseases Department, Yale School of Public Health, New Haven, Connecticut, United States of America; 2 Arnhold Institute for Global Health, Mt. Sinai School of Medicine, New York, New York, United States of America; 3 Quality Assurance Department, Uganda Ministry of Health, Kampala, Uganda; 4 Programme for Prevention and Control of Non-Communicable Diseases, Uganda Ministry of Health, Kampala, Uganda; 5 Uganda Initiative for Integrated Management of Non-Communicable Disease, Kampala, Uganda; 6 Section of General Internal Medicine, Yale School of Medicine, New Haven, Connecticut, United States of America; 7 Equity Research and Innovation Center, Yale School of Medicine, New Haven, Connecticut, United States of America; University of West London, UNITED KINGDOM

## Abstract

**Objective:**

Although the WHO-developed Service Availability and Readiness Assessment (SARA) tool is a comprehensive and widely applied survey of health facility preparedness, SARA data have not previously been used to model predictors of readiness. We sought to demonstrate that SARA data can be used to model availability of essential medicines for treating non-communicable diseases (EM-NCD).

**Methods:**

We fit a Poisson regression model using 2013 SARA data from 196 Ugandan health facilities. The outcome was total number of different EM-NCD available. Basic amenities, equipment, region, health facility type, managing authority, NCD diagnostic capacity, and range of HIV services were tested as predictor variables.

**Findings:**

In multivariate models, we found significant associations between EM-NCD availability and region, managing authority, facility type, and range of HIV services. For-profit facilities’ EM-NCD counts were 98% higher than public facilities (p < .001). General hospitals and referral health centers had 98% (p = .004) and 105% (p = .002) higher counts compared to primary health centers. Facilities in the North and East had significantly lower counts than those in the capital region (p = 0.015; p = 0.003). Offering HIV care was associated with 35% lower EM-NCD counts (p = 0.006). Offering HIV counseling and testing was associated with 57% higher counts (p = 0.048).

**Conclusion:**

We identified multiple within-country disparities in availability of EM-NCD in Uganda. Our findings can be used to identify gaps and guide distribution of limited resources. While the primary purpose of SARA is to assess and monitor health services readiness, we show that it can also be an important resource for answering complex research and policy questions requiring multivariate analysis.

## Introduction

### Background/Rationale

The World Health Organization (WHO) defines Essential Medicines (EM) as drugs considered critical to meeting the needs of the population and expects them to be accessible. To qualify as accessible, drugs must be available and affordable.[1] Yet EM used to treat non-communicable diseases (EM-NCD) remain poorly accessible to the populations of low- and middle-income countries (LMIC)[2–5], where non-communicable diseases (NCD) such as cardiovascular disease, diabetes, chronic lung disease, and mental health disorders are the leading causes of mortality. [1,6–8]

WHO has called for an 80% availability target for EM-NCD as part of a Global Action Plan, making EM-NCD a global priority.[9] However, aggregate estimates of availability at the country level may disguise stark disparities. To our understanding, the extent to which disparities for EM-NCD availability exist within individual LMIC has not previously been studied.

We sought to develop a scalable strategy for identifying within-country availability disparities from routinely collected data that could be compared across multiple LMIC. The WHO Service Availability and Readiness Assessment (SARA) is a widely endorsed methodology used to collect health facility-level data on essential medicines, technologies, and human resources.[10] This comprehensive survey of health system preparedness is intended to be performed annually and provides a national sampling of drug availability, among other indicators. At the time of publication, 11 LMIC have conducted 17 SARA surveys.[10,11] Data from SARA surveys have been used in country reports and published articles, but these have relied solely on descriptive statistics.[12–15]

In this analysis, we use SARA data to model internal disparities in the availability of EM-NCD in Uganda. Our objective was to model meaningful associations between EM-NCD availability and facility-level characteristics in a sample of Ugandan health facilities. While the primary purpose of SARA is to assess and monitor health services readiness rather than produce ready-to-analyze data for research, we show that SARA can also be an important resource for answering more complex research and policy questions using statistical methods. The objective of this analysis is not to evaluate whether or not facilities meet minimum performance expectations set out in Uganda’s Essential Medicines List (EML). Rather, the purpose is to assess the availability of a short list of EM-NCD and to identify facility characteristics associated with availability of those medicines.

## Methods

### Study design and setting

In 2013, the Ugandan Ministry of Health used the WHO SARA methodology to survey 209 health facilities in 10 districts. Healthcare in Uganda, a low-income country with a growing NCD burden[16], is delivered in three sectors: public, private-not-for-profit (PNFP), and private-for-profit (PFP). Within each sector, health facilities are divided into levels. These include health center (HC) I, II, III, IV, general hospital, and regional/national referral hospital. Each facility type varies by population served, functionality, and leadership. The HC-I level represents the community health worker program rather than facility-based services, and thus is not included in the SARA sampling.

In 2013, the Ugandan Ministry of Health, with support from WHO Country Office-Uganda, systematically sampled from facilities across these layers to conduct the SARA survey. Survey personnel visited a stratified sample of 209 Ugandan health facilities across 10 districts over a two-week period. Each health facility was assessed in one day. The presence of each medicine, equipment, or other supply was visually confirmed by the surveyor.

### Exclusions

While the complete SARA dataset for Uganda includes 13 national and regional referral hospitals, we excluded these facilities from our analysis. These referral facilities were sampled from outside the 10-district geographic frame of the other 196 facilities, which posed problems for modeling several predictor variables of interest. After excluding the referral hospitals, 196 facilities remained, including HC-II, HC-III, HC-IV, and general hospitals.

### Outcome variable

The 2013 Uganda SARA collected availability data on 20 EM, called “tracer medicines.” We identified 10 of these tracer medicines as EM-NCD. All but one of these, simvastatin, also appear on the Uganda Essential Medicines List (EML), which designates the lowest-level health facility at which each medicine is expected to be stocked (Table 1). The outcome variable, EM-NCD availability, was measured as a count score of these medicines ranging from 0 to 10. The score represents how many of the ten EM-NCD a particular facility had in stock on the day of the SARA survey.

**Table 1:** Essential medicines for treating non-communicable diseases (EM-NCD) included in the 2013 Uganda SARA survey.

Essential medicine	Disease Category	Lowest level expected	Expected facilities stocking n(%)*	Total facilities stocking n(%)*
Nifedipine cap/tab	Cardiovascular	HC-III	48 (47.1%)	64 (32.7%)
Enalapril cap/tab or alternative ACE inhibitor	Cardiovascular	Regional referral hospital	*Not expected*	33 (16.8%)
Atenolol cap/tab	Cardiovascular	Hospital	11 (64.7%)	40 (20.4%)
Metformin cap/tab	Diabetes	HC-IV	27 (79.4%)	46 (23.5%)
Glibenclamide cap/tab	Diabetes	HC-IV	26 (76.5%)	50 (25.5%)
Insulin regular	Diabetes	HC-IV	17 (50.0%)	22 (11.2%)
Salbutamol inhaler	Asthma/Chronic Obstructive Lung Disease	HC-IV	11 (32.4%)	39 (19.9%)
Beclomethasone inhaler	Asthma/Chronic Obstructive Lung Disease	HC-IV	1 (2.9%)	3 (1.5%)
Amitriptyline cap/tab	Mental health/Depression	HC-III	78 (76.5%)	93 (47.5%)
Simvastatin cap/tab	Cardiovascular	Excluded from Uganda EML	*Not expected*	6 (3.1%)

*The column labeled "Total facilities stocking" shows the total number and proportion of facilities at which each EM-NCD was available, among all 196 facilities in the sample. "Expected facilities stocking" shows the number and proportion of facilities at which each EM-NCD was available, among only those facilities at which availability is indicated by the Uganda EML.

### Independent variables

The independent variables of interest include geographic location, facility characteristics and the presence of other services or equipment. The *basic amenities domain score* for each facility is the proportion of the list of basic amenities available at a given site. The basic amenities included in the domain score were a consultation room, adequate sanitation facilities, emergency transportation, improved water source, communication equipment, power, and a computer with internet and email. Similarly, the *basic equipment domain score* is a proportion on the list of basic equipment available at a given facility. The basic equipment included in the domain score were as follows: adult scale, child scale, thermometer, stethoscope, blood pressure apparatus, and light source. Finally, *NCD diagnostic capacity* is a simple count of facility capabilities using the following tracer items: hemoglobin, blood glucose, urine dipstick (protein), urine dipstick (glucose), urine pregnancy test, and dried blood spot (DBS) collection.

If the facility offered HIV counseling and testing at the time of the survey, it was coded 1 for *HIV counseling and testing (HCT)*. If counseling and testing were not available, the facility was coded 0. Similarly, if the facility offered HIV care and support services at the time of the survey, it was coded 1 for *HIV care and support services*. If HIV care and support services were not available, the facility was coded 0.

We divided Uganda into four commonly accepted regions: West, North, East, and South. The South region includes Kampala, the capital city. We then assigned each facility to a region according to its recorded district in the SARA dataset. Because Kampala is generally acknowledged to have the greatest concentration of medical resources, we used the South region was used as the reference region.

Finally, each facility in the SARA data is identified by its managing authority, or sector. These include public, PNFP, or PFP, as defined above. In the current analysis, public facilities are the reference category to which PNFP and PFP facilities are compared. The remaining facilities were coded as HC-II, HC-III, HC-IV or General Hospital. HC-IVs offer the most services outside hospitals, while HC-II facilities offer the fewest services.

### Analysis

We fit a series of Poisson regression models using the GENMOD procedure in SAS University Edition (SAS Institute, Inc.). Beginning with a baseline model predicting NCD score by basic amenities domain score, we added independent variables hypothesized to associate with NCD score in a stepwise fashion. With the addition of each new independent variable, we assessed whether model fit was improved relative to the increased number of parameters using the Akaike information criterion (AIC). If model fit improved with the addition of a variable, we retained the variable and added the next one. Using this forward selection strategy, we reached a full “saturated” model. We then used backward elimination to remove independent variables with non-significant parameter estimates, limited contribution to model fit, or limited clinical significance until we reached our final model. All analyses were scaled to correct for over-dispersion.

To account for SARA’s complex sampling design, we weighted all our analyses using the WEIGHT option in the SAS GENMOD procedure and the sampling weights provided in the SARA dataset. Once we reached the final model, we performed diagnostics for fit and robustness with particular attention to the possibility that the SARA sampling design might result in sparse data for certain types of facilities. We checked the quality of the model fit to the data using the model deviance and degrees of freedom (see Method from SAS Proceedings Paper 247–26). Our test of the null hypothesis that there was a better fitting model than our final model returned a nonsignificant p-value, indicating that our final model was a good fit to the data. Finally, we checked the deviance and Pearson residuals for our final model and performed sensitivity analyses by removing the two observations with the greatest residuals, then assessed their impact on parameter estimates. As there was little impact, these observations were added back to the main analysis.

## Results

### Descriptive data

**Fig 1:**
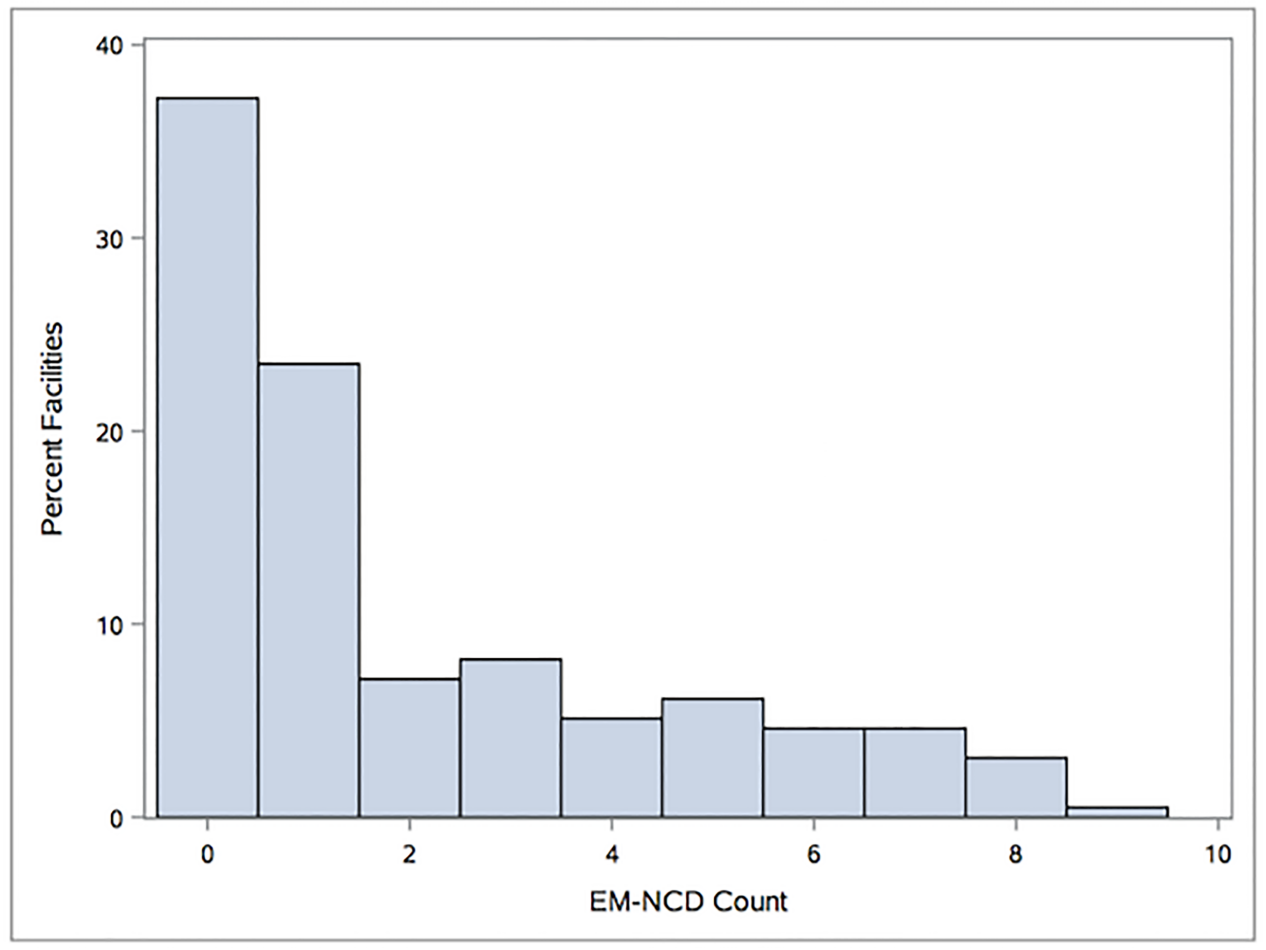
Distribution of EM-NCD counts in sampled facilities from the 2013 Uganda SARA survey.

The count of different EM-NCD present at each facility was highly skewed; scores clustered at 0, the lowest possible score, with a long tail towards 10, the highest possible score (Fig 1). More than a third of the facilities surveyed (37%) had no EM-NCD on site at all.

Table 1 describes the ten EM-NCD by category, lowest level facility expected to stock[17], and percentage of facilities stocking among the sample of facilities. No facility had all ten EM-NCD in stock. Furthermore, availability varied considerably by medicine. The least available medicine was the beclomethasone inhaler, which was only present at 3 of the 196 (1.5%) total facilities—and at only one (2.9%) of the facilities at which it was expected to be stocked. The most widely available medicine, amitriptyline, was present at a total of 93 facilities (48%), including 78 facilities at which it was expected. Presence of a given EM-NCD did not strongly correspond to the level facility at which it was expected. For example, ACE inhibitors were expected only in referral hospitals but were present at 33 facilities (17%). Conversely, injectable insulin was expected at HC-IVs and hospitals, but was only observed in 50% (17) of these facilities and in 11% (22) of all facilities.

### Main results

In bivariate analyses, region, facility type, managing authority, availability of HCT, and availability of HIV care were significantly associated with EM-NCD availability (Tables 2 and 3). In the final, preferred multivariate model (Table 4), facilities under different types of managing authority perform significantly differently in terms of EM-NCD availability. The parameter estimate for PFP facilities compared to public facilities is 0.6837; in other words, PFP facilities have EM-NCD counts that are 98% higher on average—nearly double—those of public facilities (p < .001) even after adjusting for facility level. PNFP facilities also perform significantly better than public facilities in this model, but not nearly as well as the PFP facilities. Adjusting for the other variables, PNFP facilities have average EM-NCD counts that are 47% higher on average than public facilities (p < .014).

**Table 2:** Distribution of study variables and their association with availability of NCD medicines.

Variable	N (%)	Essential medicines availability, n (%)			p*
		None present	1–3 present	4 or more present	
Region					0.04
West	23 (11.7)	12 (52.2)	5 (21.7)	6 (26.1)	
North	63 (32.1)	17 (27.0)	29 (46.0)	17 (27.0)	
East	64 (32.7)	32 (50.0)	21 (32.8)	11 (17.2)	
South	46 (23.5)	12 (26.1)	21 (45.7)	13 (28.3)	
Facility type					<0.01
General hospital	17 (8.7)	0 (0)	1 (5.9)	16 (94.1)	
HC-IV	17 (8.7)	2 (11.8)	4 (25.5)	11 (64.7)	
HC-III	68 (34.7)	6 (8.8)	50 (73.5)	12 (17.7)	
HC-II	94 (48.0)	65 (69.2)	21 (22.3)	8 (8.5)	
Managing authority					<0.01
Public	125 (63.8)	60 (48.0)	47 (37.6)	18 (14.4)	
Private non-profit	43 (21.9)	6 (14.0)	16 (37.2)	21 (48.8)	
Private for-profit	28 (14.3)	7 (25.0)	13 (46.4)	8 (28.6)	
HCT^ available	152 (77.6)	41 (27.0)	65 (42.8)	46 (30.3)	<0.01
HIV care services available	113 (57.7)	30 (26.6)	48 (42.5)	35 (31.0)	

Note: Percentages may not sum to 100% due to rounding. Frequencies and percentages reflect unweighted data.

* P-value for χ^2^ test.

^HCT = HIV Counseling and Testing

**Table 3:** Stratified distribution of study variables and their association with availability of NCD medicines.

Variable	N (%)	Essential medicines availability, n (%)			p*
		None present	1–3 present	4 or more present	
*Lower primary facilities (n = 94)*					
Region					0.02
West	14 (14.9)	12 (85.7)	0 (0.0)	2 (14.3)	
North	28 (29.8)	15 (53.6)	11 (39.3)	2 (7.1)	
East	32 (34.0)	27 (84.4)	3 (9.4)	2 (6.3)	
South	20 (21.3)	11 (55.0)	7 (35.0)	2 (10.0)	
Managing authority					<0.01
Public	61 (64.9)	54 (88.5)	6 (9.8)	1 (1.6)	
Private non-profit	14 (14.9)	5 (35.7)	6 (42.9)	3 (21.4)	
Private for-profit	19 (20.2)	6 (31.6)	9 (47.4)	4 (21.1)	
HCT^ available	53 (56.4)	33 (62.3)	12 (22.6)	8 (15.1)	0.03
HIV care available	33 (35.1)	24 (72.7)	6 (18.2)	3 (9.1)	0.78
*Primary and extended facilities (n = 102)*					
Region					0.04
West	9 (8.8)	0 (0.0)	5 (55.6)	4 (44.4)	
North	35 (34.3)	2 (5.7)	18 (51.4)	15 (42.9)	
East	32 (31.4)	5 (15.6)	18 (56.3)	9 (28.1)	
South	26 (25.5)	1 (3.9)	14 (53.9)	11 (42.3)	
Managing authority					<0.01
Public	64 (62.8)	6 (9.4)	41 (64.1)	17 (26.6)	
Private non-profit	29 (28.4)	1 (3.5)	10 (34.5)	18 (62.1)	
Private for-profit	9 (8.8)	1 (11.1)	4 (44.4)	4 (44.4)	
HCT^ available	99 (97.1)	8 (8.1)	53 (53.5)	38 (38.4)	0.84
HIV care available	80 (78.4)	6 (7.5)	42 (52.5)	32 (40.0)	

Note: Percentages may not sum to 100% due to rounding. Frequencies and percentages reflect unweighted data.

* P-value for χ^2^ test.

^HCT = HIV Counseling and Testing

**Table 4:** Poisson regression model predicting greater availability of NCD essential medicines (final/preferred model).

Variable	Adjusted β (SE)	p
Managing authority		
Public	*Reference*	---
Private non-profit	0.3882 (0.1573)	0.014
Private for-profit	0.6837 (0.1866)	< .001
Facility type		
General hospital	0.6811 (0.2372)	0.004
HC-IV	0.7154 (0.2271)	0.002
HC-III	0.2165 (0.1498)	0.148
HC-II	*Reference*	---
Region		
West	-0.0892 (0.2261)	0.693
North	-0.4217 (0.1727)	0.015
East	-0.4782 (0.1629)	0.003
South (Kampala)	*Reference*	---
Basic amenities score	1.0580 (0.3679)	0.004
Basic equipment score	1.3451 (0.4904)	0.006
NCD diagnostic capacity	0.2240 (0.0410)	< .001
HIV counseling & testing*		
Available	0.4530 (0.2295)	0.048
Not available	*Reference*	---
HIV services*		
Available	-0.4340 (0.1586)	0.006
Not available	*Reference*	---

*Dichotomous variable; reference category is 0.

The facility type parameter estimates indicate that general hospitals had EM-NCD availability scores nearly twice as high as the lowest level facilities (98% higher, p = .004). HC-IVs performed even better than general hospitals, with EM-NCD scores 105% higher than HC-II (p = .002). On average, HC-III did not have significantly greater EM-NCD availability than HC-II; these two facility types were the least likely to have any EM-NCD in stock at all.

On average, and adjusting for the other predictors, facilities in the North and East have EM-NCD availability scores 34% lower (parameter estimate = -0.4217, p = 0.015) and 38% lower (parameter estimate = -0.4782, p = 0.003), respectively, than facilities in the Kampala region.

Finally, the two dichotomous variables indicating the availability of different types of HIV-related services indicate a complex set of interrelationships between HIV/AIDS services and the availability of EM-NCD. Offering HIV care and support services was associated with 35% lower average EM-NCD counts (parameter estimate = -0.4340, p = 0.006). However, offering HIV counseling and testing was associated with 57% *higher* EM-NCD counts (parameter estimate = 0.4530, p = 0.048).

### Other analyses

Due to concerns about sparse data, we considered and rejected a zero-inflated Poisson model. While a zero-inflated model was not appropriate in the case of Uganda, researchers interested in using SARA data to analyze health systems where some types of facilities are expected to *never* have any essential medicines available should consider these types of mixed models. Other models that were considered and rejected, including a multilevel mixed model, are described in the Appendix.

## Discussion

Our findings support previous work that demonstrates that Ugandan health facilities are poorly prepared to address the growing burden of NCD.[12,16,18] We extend this previous work by identifying and quantifying clear within-country disparities in preparedness. We found significant associations between EM-NCD availability and geographic region, managing authority, health facility type, and the range of HIV services. The availability of EM-NCD was substantially higher in PFP facilities than in public facilities and strikingly lower in the North and East regions. Availability of EM-NCD had a mixed relationship to availability of care and counseling for HIV. On the one hand, facilities that offer HIV care and support had lower average EM-NCD availability. However, facilities that offer HIV counseling and testing were associated with 57% higher EM-NCD availability counts.

Our model suggests that PFP health facilities are responding most quickly to the burgeoning need for EM-NCD. Adjusting for the other variables such as facility type and amenities, PFP facilities had EM-NCD counts nearly twice as high as public facilities. This is particularly important because medicines that are subsidized and dispensed free of charge in public facilities are only available at cost in private facilities. PFP facilities are often out of financial reach for most Ugandans. For example, a controller medicine for asthma, such as beclomethasone inhaler, costs approximately seven US dollars, the equivalent of three days’ wages, based on the per capita gross domestic product.

Facility type also had a sizable effect on EM-NCD availability in our model, though the facilities offering the most sophisticated services—general hospitals—do not necessarily have the greatest availability. Adjusting for region and other facility characteristics, the HC-IV facilities outperformed even general hospitals. Primary care HC-II and HC-III facilities, on the other hand, are likely to have few, if any, EM-NCD on hand. This disparity may originate from Uganda’s hybrid “push and pull” system for supplying essential medicines in the public sector. [19,20] In this hybrid system, public sector HC-III, HC-IV, and hospitals are given discretion regarding what essential medicines they order; these facilities “pull” their essential medicine stock from the National Medical Stores. Public sector HC-IIs, however, are “pushed” a kit of essential medicines from the central stores. The “push” system can produce a mismatch between the medicines available and the needs of a specific facility.[21] In so doing, it may limit facilities’ capacity to respond to increasing demand for EM-NCD. An additional factor that influences EM availability by facility level is the Vital-Essential-Necessary (VEN) system. This stratification is embedded within the Uganda EML and adds another level of prioritization to the stocking of each medicine. Since VEN is applied to each individual EM, an assessment of its impact on availability would require a medicine-by-medicine (rather than score-based) analysis that is outside the scope of the current study but deserves attention.

It may not be surprising that facility type has a significant effect on predicted EM-NCD count. However, consistent, long-term access to these medicines is critical for the effective and uninterrupted treatment of patients with NCDs. Individual countries adapt the WHO EML based on local disease prevalence, cost-effectiveness, and other national priorities. Countries also determine the lowest-level health facilities that are expected to stock each EM (see Table 1). Based on analysis of the 2014 National Non-Communicable Disease Risk Factor Survey, 26.4% of adult Ugandans are estimated to have hypertension and there is no significant difference between urban- and rural-dwellers.[22] Given this high prevalence and limited availability of affordable medicines[23], a reanalysis of these distribution guidelines would be prudent. Limiting the supply of anti-hypertensive medicines to higher-level health facilities is incongruent with the provision of high quality, chronic care for persons with hypertension. Lower-level health facilities, where the population is expected to receive primary health care, should be expected to stock EM for NCDs such as hypertension.

There is also evidence of clear regional disparities in EM-NCD. While availability in the West region does not significantly differ from the Kampala region, facilities in the North and the East have significantly lower counts of EM-NCD than those in Kampala, even controlling for other predictors of availability. On average and adjusting for the other predictors, facilities in the North have scores 34% lower, and those in the East have scores 38% lower, than facilities in the Kampala region. One possible explanation is that the supply routes running East-West are of higher quality than those running North-South. However, in recent years, the Ugandan highway infrastructure has improved greatly and there are equally high-quality highways spanning East-West as spanning North-South. Certainly, further research is warranted towards understanding such in-country regional disparities.

Finally, the two HIV-related findings deserve special attention. We initially hypothesized that the availability of services for communicable diseases such as HIV/AIDS might be diverting resources and attention away from NCDs, resulting in lower average counts for facilities with HIV/AIDS services. However, the preferred model suggests a more complex set of interrelationships between HIV/AIDS services and the availability of EM-NCD. As hypothesized, offering HIV care and support services was associated with lower average NCD medicines counts. But offering HIV counseling and testing (HCT) was associated with *higher* counts of NCD essential medicines. It is plausible that facilities that are able to offer HCT have dispensary managers who are more attuned to the need to maintain chronic disease medicines. Or possibly these facilities have more sophisticated processes in place for monitoring and replenishing their medicine stock. Certainly, this is a result that we find compelling and in need of further study.

SARA data are collected using a complex, non-representative sampling strategy that must be corrected for using sample weights. In addition, SARA sample sizes are neither intentionally, nor necessarily, powered to provide significant estimates in regression models. This has been an impediment to wider use of these important data. Both the openly available country SARA reports and all prior published research using SARA data have relied only on descriptive statistics, reporting simple unadjusted proportions rather than associations. We have shown that, despite these perceived barriers, researchers can use SARA data to develop regression models by applying straightforward corrections and diagnostic checks. By conducting the first Poisson analysis using SARA data, we have identified multiple disparities in availability of EM-NCD within Uganda.

Our approach had some limitations. First, like any cross-sectional design, ours is unable to infer causality. Longitudinal research is needed to better understand the sources of availability disparities like those we describe. Second, the most accurate level designation for private facilities is open to some interpretation, as these designations originate from the public sector. We used all level designations for facility as recorded in the SARA dataset. However, private sector facilities, specifically PFP, regardless of their MOH designation level, are sensitive to economic forces such as client demand and, thus, are likely to perform at levels higher than public sector facilities of the same level. This misalignment within facilities of the same level represents a potential limitation. Third, the SARA tool does not collect data on EM cost, thereby limiting its utility for directly addressing access, which is a function of both availability and cost. Fourth, like other EM availability surveys, SARA data reflect stock on the pharmacy shelf on a single day. This approach fails to account for variability in stock over time, which could be substantial and might particularly influence estimates of geographic disparity. Finally, though the public-facing data summary was available via the WHO[24], obtaining the raw dataset for analysis was challenging. These limitations point to the unmet need for technologies that provide real-time, hyper-local data to help spotlight and redress disparities in access faster—and to map, measure, and monitor disparities in access to care. Overlaying such insights with disease prevalence, population density, and health determinants such as traffic patterns and household income would further increase utility for decision-makers.

To deepen our understanding of variation in EM-NCD availability within LMIC, future research should aim to understand facility- and system-level barriers and facilitators to EM-NCD availability. As more LMIC conduct SARA surveys, these datasets represent a largely untapped empirical resource for global health researchers and policymakers. We demonstrate that data generated by the SARA tool may be used to generate a robust, informative statistical model by applying well-recognized techniques to correct for some of the most common challenges inherent in these data. The results of such analyses can guide operational research and inform decision-making, investment, and priority-setting.

## Supplemental analyses

Given the complex sampling strategy and the possibility that health facilities in the same district may influence one another with regard to availability of EM-NCD, we also fit a multilevel mixed model to supplement our primary analysis. There was little evidence of need for a multilevel model and the parameter estimates of the multilevel mixed model were in general agreement with those of the easier-to-interpret Poisson model presented in the main analysis.

We also considered an alternative model including the presence of *other* essential medicines as a predictor, which was rejected because of evidence of serious multicollinearity.
